# Exon-intron structure and sequence variation of the calreticulin gene among *Rhipicephalus sanguineus* group ticks

**DOI:** 10.1186/s13071-016-1909-3

**Published:** 2016-12-12

**Authors:** Daniele Porretta, Maria Stefania Latrofa, Filipe Dantas-Torres, Valentina Mastrantonio, Roberta Iatta, Domenico Otranto, Sandra Urbanelli

**Affiliations:** 1 Department of Environmental Biology, Sapienza University of Rome, Rome, Italy; 2 Department of Veterinary Medicine, University of Bari, 70010 Valenzano, Bari Italy; 3 Department of Immunology, Aggeu Magalhães Research Centre, Oswaldo Cruz Foundation, 50740465 Recife, PE Brazil

**Keywords:** Hard tick, *Rhipicephalus sanguineus* group, Intron evolution, Intron presence-absence polymorphism, Tick control, Genetic markers

## Abstract

**Background:**

Calreticulin proteins (CRTs) are important components of tick saliva, which is involved in the blood meal success, pathogen transmission and host allergic responses. The characterization of the genes encoding for salivary proteins, such as CRTs, is pivotal to understand the mechanisms of tick-host interaction during blood meal and to develop tick control strategies based on their inhibition. In hard ticks, *crt* genes were shown to have only one intron with conserved position among species. In this study we investigated the exon-intron structure and variation of the *crt* gene in *Rhipicephalus* spp. ticks in order to assess the *crt* exon-intron structure and the potential utility of *crt* gene as a molecular marker.

**Methods:**

We sequenced the exon-intron region of *crt* gene in ticks belonging to so-called tropical and temperate lineages of *Rhipicephalus sanguineus* (*sensu lato*), *Rhipicephalus* sp. I, *Rhipicephalus* sp. III, *Rhipicephalus* sp. IV, *R. guilhoni*, *R. muhsamae* and *R. turanicus*. Genetic divergence and phylogenetic relationships between the sequences obtained were estimated.

**Results:**

All individuals belonging to the tropical lineage of *R. sanguineus* (*s.l.*), *R. guilhoni*, *R. muhsamae*, *R. turanicus*, *Rhipicephalus* sp. III and *Rhipicephalus* sp. IV analysed showed *crt* intron-present alleles. However, both *crt* intron-present and intron-absent alleles were found in *Rhipicephalus* sp. I and the temperate lineage of *R. sanguineus* (*s.l.*), showing the occurrence of an intraspecific intron presence-absence polymorphism. Phylogenetic relationships among the *crt* intron-present sequences showed distinct lineages for all taxa, with the tropical and temperate lineages of *R. sanguineus* (*s.l.*) being more closely related to each other.

**Conclusions:**

We expanded previous studies about the characterization of *crt* gene in hard ticks. Our results highlighted a previously overlooked variation in the *crt* structure among *Rhipicephalus* spp., and among hard ticks in general. Notably, the intron presence/absence polymorphism observed herein can be a candidate study-system to investigate the early stages of intron gain/loss before fixation at species level and some debated questions about intron evolution. Finally, the sequence variation observed supports the suitability of the *crt* gene for molecular recognition of *Rhipicephalus* spp. and for phylogenetic studies in association with other markers.

**Electronic supplementary material:**

The online version of this article (doi:10.1186/s13071-016-1909-3) contains supplementary material, which is available to authorized users.

## Background

Calreticulins (CRTs) are calcium-binding proteins that have been found in a broad range of eukaryotic organisms, both invertebrates and vertebrates [1–3]. They can be implicated in many cellular functions, including cell proliferation, calcium storage, protein folding, modulation of gene expression, cell apoptosis and cell differentiation [4–6].

In ticks, CRTs are important components of tick saliva, which plays a pivotal role in tick feeding and pathogen transmission to hosts by acting as anti-haemostatic, anti-inflammatory and immunomodulatory molecules [7–15]. In this context, CRTs have been suggested to play a modulating role in host haemostasis [9, 16, 17] and have been shown to be highly immunogenic to tick mammalian hosts [18–21]. These findings have fuelled the interest for CRTs, as they could be used as targets for diagnostic tools (e.g. for detecting exposure to tick bites) and vaccines [22]. Several studies were therefore focused on the genetic characterization of CRTs and to date *crt* genes have been sequenced in several hard tick species [23–26]. Comparative analysis of the exon-intron structure of *crt* genes in 28 hard tick species belonging to different genera, including *Amblyomma*, *Dermacentor*, *Ixodes* and *Rhipicephalus* showed that (i) two exons and only one intron are present in tick *crt* genes, contrary to what was observed in other invertebrate and vertebrate species (i.e. two introns in nematodes, three in fruit flies, seven in mouse and eight in humans); (ii) the intron position is conserved in hard ticks, although the intron size and nucleotide sequences vary among species [24, 25]. Noteworthy, the *crt* gene has been found to be expressed in salivary glands and in several other tissues (i.e. cuticle, gut, fat body, ovaries) and at different tick developmental stages [24, 26], where CRTs are likely involved in multiple cellular functions as described above [4]. *Crt* genes could be therefore good target sites for control strategies based on gene expression silencing approaches such as RNA interference (RNAi), as their silencing could not only affect the success of tick blood feeding and pathogen transmission but also tick physiology and fitness [27, 28].

In addition, *crt* are single-copy genes [4] and the occurrence of a conserved intron-exon structure makes them a potential good exon-primed intron-crossing (EPIC) marker to be used in phylogenetic studies among closely related taxa [29–32]. EPIC, having both the exon and intron fragments, could help in examining genetic variation at the intraspecific and interspecific level simultaneously, which could be particularly helpful when studying a species complex [29, 30, 32].

Among ticks, the *Rhipicephalus sanguineus* group is particularly important from medical and veterinary viewpoints, as its members are vectors of several tick-borne pathogens causing diseases in dogs (e.g. *Ehrlichia canis*, *Babesia vogeli* and *Hepatozoon canis*) and humans (e.g. *Rickettsia conorii* and *Rickettsia rickettsii*) [33–35]. In the last two decades, ecological, morphological and genetic studies have concordantly supported the occurrence of distinct cryptic species within this group, whose geographic distribution, taxonomic status and phylogenetic relationships are still debated [36–39].

To date, *crt* gene sequence has been obtained only from a single individual of *R. sanguineus* (*sensu lato*) from Colombia [24, 25]. Indeed, sequence diversity and structure of the *crt* gene have been poorly investigated within and among members of the *R. sanguineus* group. In the context, we aimed to contribute to address this gap by analysing the *crt* gene in different *Rhipicephalus* spp., including *R. sanguineus* (*s.l.*), *R. turanicus*, *R. guilhoni*, *R. muhsamae* and additional Operational Taxonomic Units (OTUs) that have been recently identified on the basis of morphological and molecular analysis [40]. Therefore, the *crt* exon-intron region from each individual was sequenced and analysed among species and the OTUs listed above. The ability of the *crt* gene sequence to correctly identify the individuals analysed was also tested to assess its potential utility as a molecular marker for *Rhipicephalus* spp.

## Methods

### Ticks

Ticks belonging to the *R. sanguineus* group used in this study are a subset of the ticks morphologically and genetically identified in the study of Dantas-Torres et al. [40]. They include individuals of *R. sanguineus* (*s.l.*) (= “tropical species”), *Rhipicephalus* sp. I, *Rhipicephalus* sp. II (= “temperate species”), *Rhipicephalus* sp. III, *Rhipicephalus* sp. IV, *R. turanicus* and *R. guilhoni*. For clarity’s sake, ticks referred to as “*R. sanguineus* (*s.l.*)” and “*Rhipicephalus* sp. II” in Dantas-Torres et al. [40] will be referred here as tropical and temperate lineages, respectively, as these designations have been consensually used in the literature. Taxa with at least three individuals each and from different geographic areas were selected (Table 1). One *R. muhsamae* individual, which does not belong to the *R. sanguineus* group, was also included. The genomic DNA of each tick, previously extracted [40], was used for amplification and sequencing of the *crt* gene.

**Table 1:** *Rhipicephalus* spp. ticks analysed. Individuals with the same Genbank accession number share the same haplotype

Species	Code	Geographical origin	Intron	5′ splice donor	Intron size (bp)	3′ splice donor	GenBank accession number
*R. sanguineus* ^a^	–	Colombia	yes	GGAG/gtgagta	341	gtgcag/ATGC	AY395275
Tropical lineage	tick224	Vietnam (Ho Chi Minh City)	yes	GGAG/gtgagta	338	gtgcag/ATGC	KX951737
	tick228	Thailand (Bangkok)	yes	GGAG/gtgagta	338	gtgcag/ATGC	KX951737
	tick230	Thailand (Bangkok)	yes	GGAG/gtgagta	338	gtgcag/ATGC	KX951738
	tick249	Honduras (San Pedro)	yes	GGAG/gtgagta	338	gtgcag/ATGC	KX951746
	tick250	Honduras (San Pedro)	yes	GGAG/gtgagta	338	gtgcag/ATGC	KX951737
	tick259	Costa Rica (San Jose)	yes	GGAG/gtgagta	340	gtgcag/ATGC	KX951746
	tick260	Costa Rica (San Jose)	yes	GGAG/gtgagta	340	gtgcag/ATGC	KX951746
*Rhipicephalus* sp. I	tick129	Italy (Putignano)	no	GGAG/gtgagta	–	gtgcag/ATGC	KX951751
	tick130	Italy (Putignano)	no	GGAG/gtgagta	–	gtgcag/ATGC	KX951751
	tick131	Italy (Putignano)	no	GGAG/gtgagta	–	gtgcag/ATGC	KX951751
	tick137	Italy (Putignano)	no	GGAG/gtgagta	–	gtgcag/ATGC	KX951751
	tick68	Greece (Xanthi)	yes	GGAG/gtgagta	339	gtgcag/ATGC	KX951747
	tick68	Greece (Xanthi)	no	GGAG/gtgagta	–	gtgcag/ATGC	KX951751
	tick73	Greece (Xanthi)	yes	GGAG/gtgagta	338	gtgcag/ATGC	KX951739
	tick269	Greece (Xanthi)	no	GGAG/gtgagta	–	gtgcag/ATGC	KX951751
	tick270	Greece (Xanthi)	no	GGAG/gtgagta	–	gtgcag/ATGC	KX951751
	tick274	Greece (Xanthi)	no	GGAG/gtgagta	–	gtgcag/ATGC	KX951751
	tick278	Greece (Xanthi)	no	GGAG/gtgagta	–	gtgcag/ATGC	KX951751
Temperate lineage	tick28	Spain (La Vera, Santa Cruz de Tenerife)	yes	GGAG/gtgagta	338	gtgcag/ATGC	KX951740
	tick32	Spain (La Vera, Santa Cruz de Tenerife)	yes	GGAG/gtgagta	338	gtgcag/ATGC	KX951740
	tick216	Portugal	yes	GGAG/gtgagta	338	gtgcag/ATGC	KX951741
	tick210	Italy (Messina)	no	GGAG/gtgagta	–	gtgcag/ATGC	KX951751
	tick211	Italy (Messina)	no	GGAG/gtgagta	–	gtgcag/ATGC	KX951751
*Rhipicephalus* sp. III	tick196	Pakistan (Punjab)	yes	GGAG/gtgagta	338	gcgcag/ATGC	KX951742
*Rhipicephalus* sp. IV	tick144	Nigeria (Plateau State)	yes	GGAG/gtgagta	336	gtgcag/ATGC	KX951748
	tick 145	Nigeria (Plateau State)	yes	GGAG/gtgagta	336	gtgcag/ATGC	KX951748
*R. turanicus*	tick153	Italy (Accettura)	yes	GGAG/gtgagta	335	gtgcag/ATGC	KX951743
	tick154	Italy (Accettura)	yes	GGAG/gtgagta	335	gtgcag/ATGC	KX951743
	tick155	Italy (Accettura)	yes	GGAG/gtgagta	335	gtgcag/ATGC	KX951744
	tick156	Italy (Accettura)	yes	GGAG/gtgagta	335	gtgcag/ATGC	KX951745
	tick157	Italy (Accettura)	yes	GGAG/gtgagta	335	gtgcag/ATGC	KX951744
*R. guilhoni*	tick140	Nigeria (Plateau State)	yes	GGAG/gtgagta	335	gtgcag/ATGC	KX951750
	tick141	Nigeria (Plateau State)	yes	GGAG/gtgagta	335	gtgcag/ATGC	KX951750
	tick142	Nigeria (Plateau State)	yes	GGAG/gtgagta	335	gtgcag/ATGC	KX951750
*R. muhsamae*	tick148	Nigeria (Plateau State)	yes	GGAG/gtgagta	332	gtgcag/ATGC	KX951749

^a^Sequence from Xu et al. [25]

### PCR amplification and sequencing

We initially amplified and sequenced a *crt* fragment that includes the intron region using the degenerate primers pair CRT32DF 5'-ATG CGG STY STS TGC WTK TTG C-3′; CRT1268DRC 5′-CTC AMA RYT CYT CGT GST YGT G-3′ [25]. Then a new primer pair (*Rsang-crt*-F 5′-CAT TTT GCT TCC CCT GGT-3′; *Rsang-crt*-R 5′-TGT TCT GTT CGT GCT TGA-3′) was designed within the DNA sequence obtained to increase the specificity of the amplification and used for further analyses of *Rhipicephalus* ticks. On the basis of the position of the PCR primers designed on the *R. sanguineus crt* sequence available in GenBank, PCR amplicons of about 630 bp were expected [25]. Each PCR amplification was performed in 25 μl including 1× buffer, 200 mM dNTPs, 2.5 mM MgCl_2_, primers at 0.2 mM, 0.5 units of high fidelity Taq DNA Polymerase (PhusionH High-Fidelity DNA Polymerase, Fermentas-Thermo Scientific Life Science, Milan, Italy), and 20 ng of genomic DNA. Negative controls containing all reagents and water instead of DNA, were included in all PCR amplifications to check for contamination. The PCR cycling procedure was: 95 °C for 5 min followed by 34 cycles at 93 °C for 1 min, 57 °C for 1 min, 72 °C for 1 min 30 s, and a single final step at 72 °C for 10 min.

PCR products were run on 1% agarose, 0.5× TAE electrophoresis gel, and visualized by staining with Gelred (Sigma-Aldrich, Milan, Italy). The sizes of the DNA fragments were assessed using the 100 bp DNA ladder (Promega, Milan, Italy) run on the same gel. After electrophoresis, PCR products were purified using the NucleoSpin Gel and PCR Cleanup kit (Macherey-Nagel, Carlo Erba, Milan, Italy) following the manufacturer’s protocol. Two PCR bands of about 630 and 300 bp, respectively, were observed by running the PCR amplicons of the *Rhipicephalus* sp. I tick68 individual (see Results section). Both bands were then excised by gel and purified as described above.

PCR products were sequenced using ABI PRISM 3700 DNA sequencer by Macrogen Inc. (www.macrogen.com). All individuals were double sequenced using both forward and reverse primers to check for consistency and all *crt* sequences that were found unique were re-amplified and re-sequenced. Sequences were edited and aligned using the software Chromas 2.31 (Technelysium Pty Ltd, Australia) and Clustal X 2.1 [41], respectively. Polymorphisms of nucleotide and amino-acidic sequences were assessed using the software DnaSP 5.10.1 [42]. Pairwise p-distances between the *crt* sequences obtained were computed using MEGA 7.0 [43]. The genealogical relationships between *crt* sequences were investigated by constructing a phylogenetic network using the median-joining (MJ) network algorithm as implemented in the NETWORK 5.0.0 software (Fluxus Technology Ltd). The loops in the resulting phylogenetic network were resolved by applying the criteria described by Pfenninger and Posada [44]. The *R. sanguineus* (*s.l.*) *crt* gene sequence AY395275 [25] was included in the analyses for comparison.

## Results

**Fig. 1:**
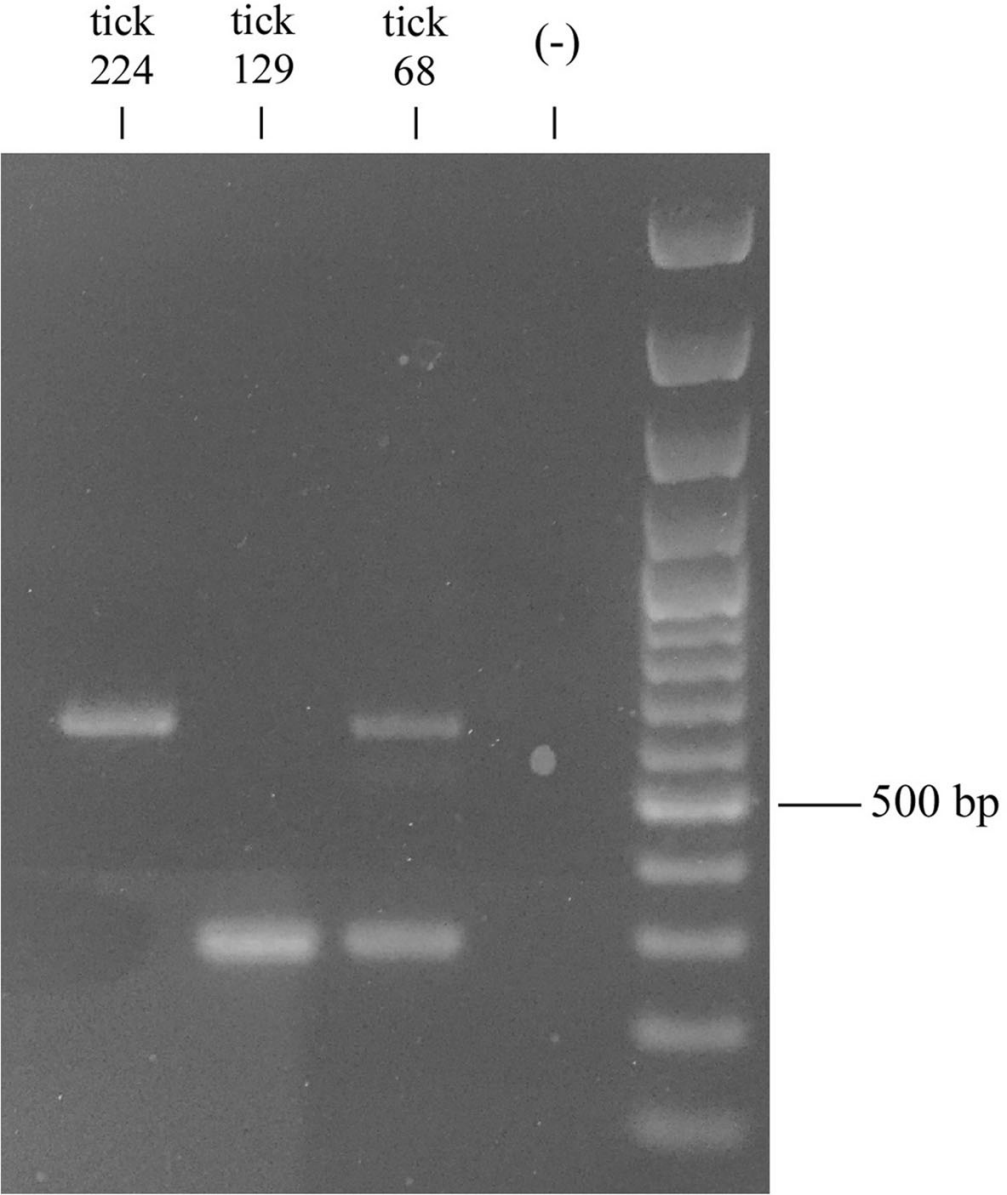
Electrophoretic pattern of calreticulin PCR products on 1% agarose. Lanes 1–3: PCR products of *Rhipicephalus* spp. individuals (codes as in Table 1); Lane 4: PCR negative control; Lane 5: 100 bp DNA ladder (Promega, Milan, Italy)

Successful PCR amplifications were obtained from 35 individuals. Among them, 24 showed PCR amplicons of the expected size, while ten individuals showed amplicons of about 300 bp (two belonging to the temperate lineage and eight *Rhipicephalus* sp. I) and one individual (*Rhipicephalus.* sp. I tick68) showed PCR amplicons of both sizes (Table 1, Fig. 1).

All PCR amplicons were sequenced and the sequences obtained were deposited in GenBank (accession numbers KX951737–KX951751) (Table 1). By comparing the obtained sequences with the genomic *crt* sequence of *R. sanguineus* (*s.l.*) from Colombia available in GenBank (AY395275), we found nucleotide identity ranging from 95% (*R. muhsamae* tick148) to 99% (*R. sanguineus* (*s.l.*) tick224), showing that all sequences obtained corresponded to the target *crt* gene region. No double peaks were found in the sequence chromatograms, showing that no heterozygous individuals were found.

**Fig. 2:**
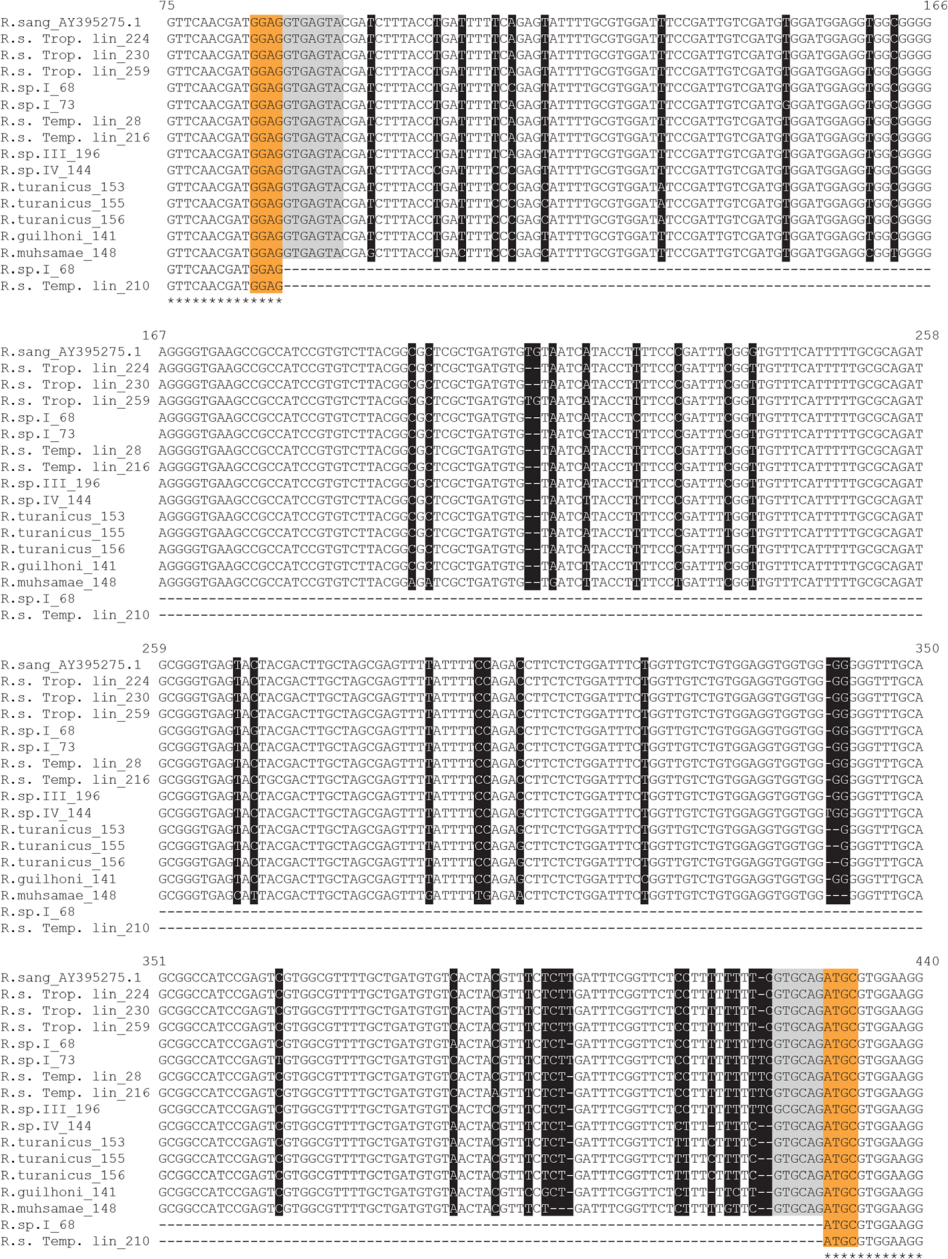
Alignment of *crt* exon-intron region in *Rhipicephalus* spp. individuals analysed (codes as in Table 1). Nucleotides in orange boxes belong to exon regions in 5′ and 3′ splice donor regions. Nucleotides in light grey boxes belong to intron region in 5′ and 3′ splice donor regions. Nucleotides in black boxes indicate sequence identity in intron region. Asterisks show nucleotide identity in exon regions. Numbers indicate the nucleotide position of the *R. sanguineus* (*s.l*.) *crt* sequence AY395275.1 [25]. *Abbreviations*: R.s. Trop. lin., *Rhipicephalus sanguineus* (*s.l*.) “Tropical lineage”; R.s. Temp. lin., *Rhipicephalus sanguineus* (*s.l*.) “Temperate lineage”

The longer *crt* fragments had the expected exon-intron structure, including two fragments of exon regions and one intron region of 332–340 bp (Table 1, Fig. 2, Additional file 1: Figure S1). *Crt* exon-intron structure was found in all individuals belonging to the tropical lineage, *Rhipicephalus* sp. III, *Rhipicephalus* sp. IV, *R. turanicus*, *R. guilhoni* and *R. muhsamae* analysed, as well as in three out of the five temperate lineage individuals and in two out of the 11 *Rhipicephalus* sp. I individuals analysed (Table 1). Fourteen unique sequences were identified by 49 polymorphic sites (11 sites in the exon regions with eight synonymous and three non-synonymous changes) (Additional file 1: Figure S1).

The mean pairwise *p*-distance between all *crt*-intron sequences was 2% (standard error, SE = 0.3%; range = 0.2–4.3%). Mean pairwise *crt* divergence within taxa was 0.3% (SE = 0.2%) for tropical lineage*,* 1.4% (SE = 0.5%) for *Rhipicephalus* sp. I, 0.5% (SE = 0.3%) for *Rhipicephalus* sp. II, and 0.2% (SE = 0.2%) for *R. turanicus* individuals. Mean pairwise divergence between taxa ranged from 0.3% (SE = 0.2%) (between tropical and temperate lineages) and 4.1% (SE = 0.8%) (between *Rhipicephalus* sp. III and *R. muhsamae*) (Table 2).

**Table 2:** Mean pairwise genetic distance between the *Rhipicephalus* spp. analysed for the calreticulin gene sequences. Ticks are encoded as in Table 1. Mean *p*-distances (below the diagonal) have been estimated as implemented in the MEGA 7.0 software. Only sequences with intron region were used. Standard error estimates are shown above the diagonal and were obtained by a bootstrap procedure (1,000 replicates)

	Tropical lineage	*R.* sp. I	Temperate lineage	*R.* sp. III	*R.* sp. IV	*R. turanicus*	*R. guilhoni*	*R. muhsamae*
Tropical lineage	–	0.003	0.002	0.003	0.005	0.006	0.007	0.007
*Rhipicephalus* sp. I	0.009	–	0.003	0.004	0.005	0.006	0.007	0.007
Temperate lineage	0.003	0.009	–	0.004	0.005	0.006	0.007	0.007
*Rhipicephalus* sp. III	0.008	0.014	0.009	–	0.006	0.007	0.007	0.008
*Rhipicephalus* sp. IV	0.018	0.018	0.018	0.022	–	0.005	0.005	0.007
*R. turanicus*	0.025	0.027	0.026	0.027	0.015	–	0.006	0.007
*R. guilhoni*	0.027	0.028	0.028	0.029	0.017	0.023	–	0.008
*R. muhsamae*	0.039	0.039	0.040	0.041	0.034	0.039	0.039	–

The genealogical relationships between the *crt*-intron alleles are shown in the median-joining network reported in Fig. 2. Distinct lineages were observed for *R. turanicus*, *R. guilhoni*, *R. muhsamae*, *Rhipicephalus* sp. III and *Rhipicephalus* sp. IV. Individuals belonging to the tropical and temperate lineages were more closely related to each other, but showed distinct alleles. The *Rhipicephalus* sp. I tick73 allele derived from the *Rhipicephalus* sp. II tick28 allele, while the *Rhipicephalus* sp. I tick68 allele derived from a missing intermediate allele.

Contrary to the *crt-*intron fragments, the smaller *crt* fragments did not show the expected intron region. These *crt* intron-absent alleles were all identical (Fig. 2; Additional file 1: Figure S1) across ten individuals among those analysed: all *Rhipicephalus* sp. I ticks with the exception of the *Rhipicephalus* sp. I tick73, that showed a *crt* intron-present allele, and the *Rhipicephalus* sp. I tick68, that showed both intron-present and intron-absent alleles (Table 1, Figs. 1 and 2); two temperate lineage individuals (*Rhipicephalus* sp. II tick210, *Rhipicephalus* sp. II tick211) (Table 1).

## Discussion

Sequence structure and diversity of the *crt* gene have been poorly investigated in *Rhipicephalus* spp. ticks [24, 25]. In this paper, our first aim was to assess if the *crt* gene structure is conserved among *R. sanguineus* group taxa and other species, such as *R. muhsamae*. Previous analyses of the *crt* gene structure in 28 hard tick species (one individual per species) belonging to seven genera (*Amblyomma, Boophilus*, *Dermacentor*, *Hyalomma*, *Haemaphysalis*, *Ixodes* and *Rhipicephalus*) showed that *crt* gene has only one intron and that its position is conserved [24, 25]. By sequencing the *crt* exon-intron region from eight species/OTUs of *Rhipicephalus*, we showed the occurrence of *crt* intron-present alleles in all tropical lineage, *R. turanicus*, *R. guilhoni*, *Rhipicephalus* sp. III, *Rhipicephalus* sp. IV and *R. muhsamae* individuals, from different geographic regions (Table 1), supporting the occurrence of an intron in the *crt* gene at the expected position [24, 25]. Surprisingly, by analysing ticks belonging to *Rhipicephalus* sp. I and temperate lineage, we found both *crt* intron-present and intron-absent alleles (Table 1). Therefore, our results, first, supported the conserved position of the intron region in the *crt* gene as shown by Xu et al. [25]; second, they highlighted a previously overlooked variation in the *crt* exon-intron structure among *Rhipicephalus* spp., and among hard ticks in general.

Introns are a common feature of the eukaryote genomes [45, 46]. The intron density can vary by more than three orders of magnitude among genomes of different organisms. Different numbers of introns in homologous genes have been documented for several closely or distantly related taxa [47]. On the contrary, only few cases of intra-specific variation (i.e. intron presence-absence polymorphism within or among populations of the same species) have been documented to date [48–50]. In arthropods, it has been found in *Drosophila melanogaster* populations at the locus *4f-rnp* [48] and in *Drosophila teissieri*, where the intron presence-absence polymorphism observed at the *jingwei* gene was ascribed to the action of positive selection [49]. The *crt* gene in *R. sanguineus* group adds to these rare cases. Indeed, both *crt* intron-present and intron-absent alleles were found in *Rhipicephalus* sp. I and temperate lineage ticks, and even both alleles were found within the same population of *Rhipicephalus* sp. I (i.e. Xanthi, Greece and Putignano, Italy) (Table 1). The *crt* gene in *Rhipicephalus* spp. is therefore a candidate study-system to investigate the early stages of intron gain/loss before fixation at the species level, as well as to investigate the evolutionary and ecological factors underlying their persistence and diffusion. In this context, because introns can significantly affect gene expression [45, 51], future studies are warranted to investigate the phenotypic effects of the intron presence/absence polymorphism observed (i.e. *crt* gene expression and, ultimately, the tick blood meal success) in order to assess its potential adaptive value.

**Fig. 3:**
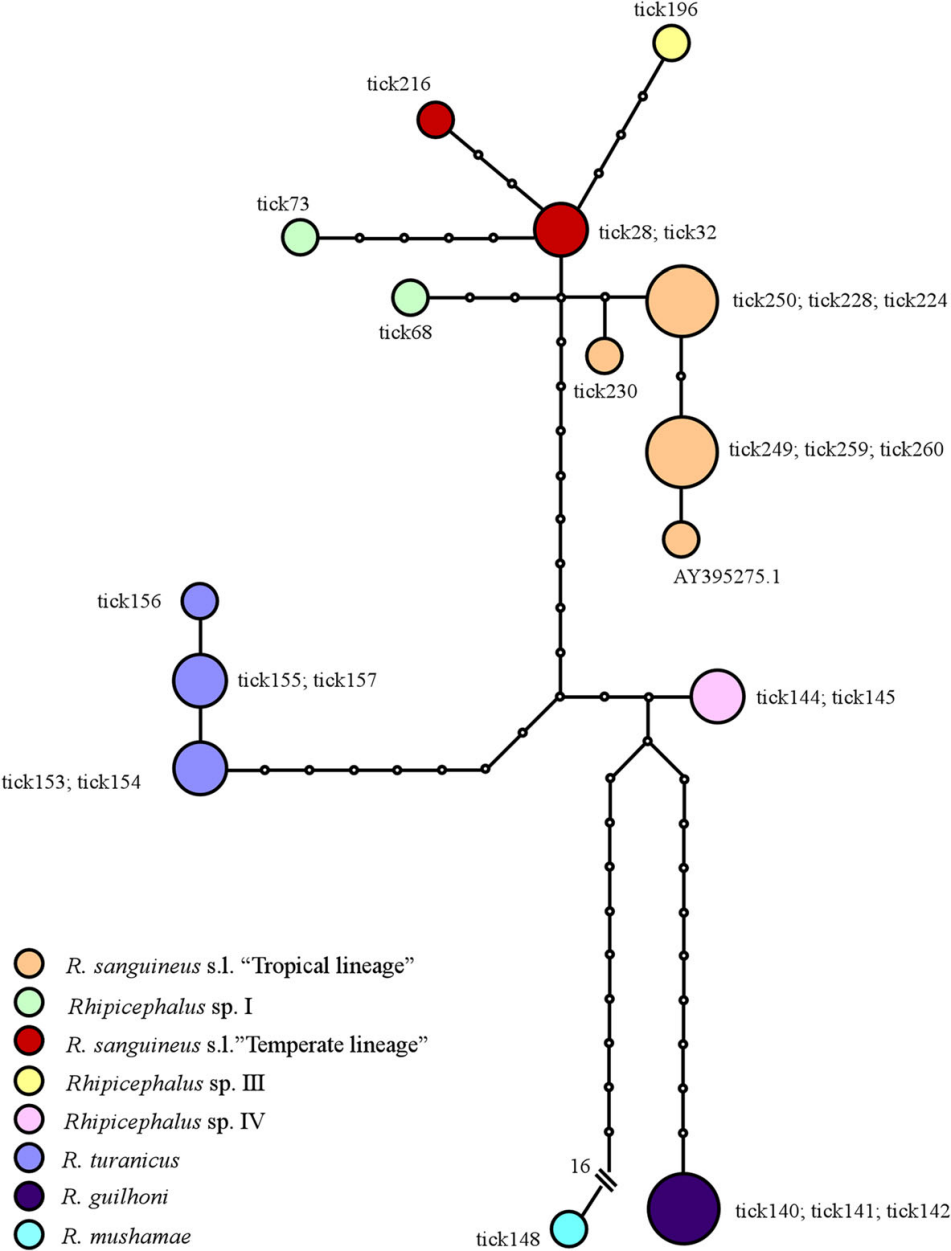
Median-joining network showing phylogenetic relationships among the *crt*-intron sequences of the *Rhipicephalus* spp. ticks analysed. *Crt* alleles are shown as circles with sizes corresponding to their frequencies in the total sample and colour corresponding to *Rhipicephalus* taxon where they have been observed. Alleles are coded as in Table 1. Dots indicate missing intermediate alleles

In addition, the potential utility of the *crt* gene as molecular marker has been herein assessed. Taxonomic status and phylogenetic relationships between the members of the *R. sanguineus* group are still debated [37]. Mitochondrial DNA (mtDNA) regions such as 16S and 12S ribosomal DNA (rDNA) and cytochrome *c* oxidase subunit 1 (*cox*1) gene, have been widely used as genetic markers that have proven to be useful in identifying cryptic diversity within the *R. sanguineus* group [36, 38–40, 52, 53]. However, because the mtDNA genome is inherited as a unit, mitochondrial genes cannot be regarded as independent sources of phylogenetic information. Furthermore, single loci are subject to issues of non-concordance between gene and species trees due to introgression, incomplete lineage sorting, natural selection and arbitrary divergence that could obscure the real population structure [54–57]. Multilocus analysis, using both mtDNA and nuclear DNA markers, could overcome the above drawbacks. Among nuclear markers, the internal transcribed spacer-2 (ITS-2) has been used in some studies, but little interspecific divergence was found and this marker was unable to distinguish between *Rhipicephalus* spp., such as between the tropical lineage and *R. turanicus* [58, 59] or between the tropical lineage, *R. guilhoni* and *R. turanicus* [36]. The polymorphism observed at the *crt* gene fragment analysed, concordantly with mtDNA markers [40], showed that *R. muhsamae* is the most distantly related taxon among those analysed (Fig. 3, Table 2). The above findings support the suitability of the *crt* gene for rapid molecular recognition of *R. sanguineus* group taxa that ITS-2 region failed to identify, as well as its potential utility for phylogenetic studies in association with other markers.

From a taxonomic point of view, of particular interest are the findings that *Rhipicephalus* sp. I and the temperate lineage have both *crt* intron-present and intron-absent alleles (Table 1). These two taxa have been recently described and characterized by morphological and molecular analyses (variation at the mtDNA loci 12S rDNA, 16S rDNA and *cox*1) [40]. Under the *Rhipicephalus* sp. I were included ticks from southern Italy and Greece, while the so-called temperate lineage comprised ticks from temperate regions of the Americas and Europe [40]. The observed sharing of *crt* intron-present and intron-absent alleles between *Rhipicephalus* sp. I and the temperate lineage could be due to current gene exchange. Interestingly, the *Rhipicephalus* sp. I tick68 showed both alleles, which could support that this taxon has a heterozygous genotype at this locus. Alternatively, reproductive isolation could be actually completed and the observed pattern could be the signature of past hybridization events or incomplete lineage sorting [54–56]. Crossbreeding experiments as well as the analysis of *Rhipicephalus* sp. I and temperate lineage populations may help to test these hypotheses.

## Conclusions

The tick saliva, as in other haematophagous arthropods, is involved in the success of blood feeding, in pathogen transmission and host allergic responses. The characterization of the genes encoding for the salivary molecular components, such as CRTs, is pivotal to understand the mechanisms of tick-host interaction during the blood meal as well as to develop tick control strategies based on their inhibition [7, 9, 13, 16, 17]. In this study we focused on the *R. sanguineus* group and expanded previous studies about the characterization of *crt* gene in hard ticks. The unexpected finding of intron presence-absence polymorphism in the *crt* gene within and among *Rhipicephalus* spp. opens some questions about how widespread is this polymorphism among hard ticks and its evolutionary significance. What are the phenotypic effects of the intron presence/absence in *crt* gene and how intron absence can affect tick fitness and tick-host interactions? What are the evolutionary processes that underlie and maintain the sharing of intron-present and intron-absent alleles in *Rhipicephalus* sp. I and in the temperate lineage? The answers to these questions will contribute not only to our understanding about the biology and ecology of the *R. sanguineus* group ticks, but also may help address some unresolved questions about the evolution of eukaryotic genes.

## Additional file

Additional file 1:
**Figure S1.** Alignment of the full sequence of the *crt* region amplified in the *Rhipicephalus* spp. individuals analysed (coded as in Table 1). Nucleotides in orange boxes belong to exon regions in 5′ and 3′ splice donor regions. Nucleotides in light grey boxes belong to intron region in 5′ and 3′ splice donor regions. Nucleotides in black boxes indicate sequence identity in intron region. Asterisks show nucleotide identity in exon regions. Numbers indicate the nucleotide position of the *R. sanguineus* (*s.l.*) *crt* sequence AY395275 [25]. *Abbreviations*: R.s. Trop. lin., *Rhipicephalus sanguineus* (*s.l*.) “Tropical lineage”; R.s. Temp. lin., *Rhipicephalus sanguineus* (*s.l*.) “Temperate lineage”. (TIF 1069 kb)
