# Supplementary figures and images for: Exon-intron structure and sequence variation of the calreticulin gene among Rhipicephalus sanguineus group ticks
Source: Parasit Vectors. 2016 Dec 12;9:640. doi: 10.1186/s13071-016-1909-3 (PMC5154033; doi:10.1186/s13071-016-1909-3)

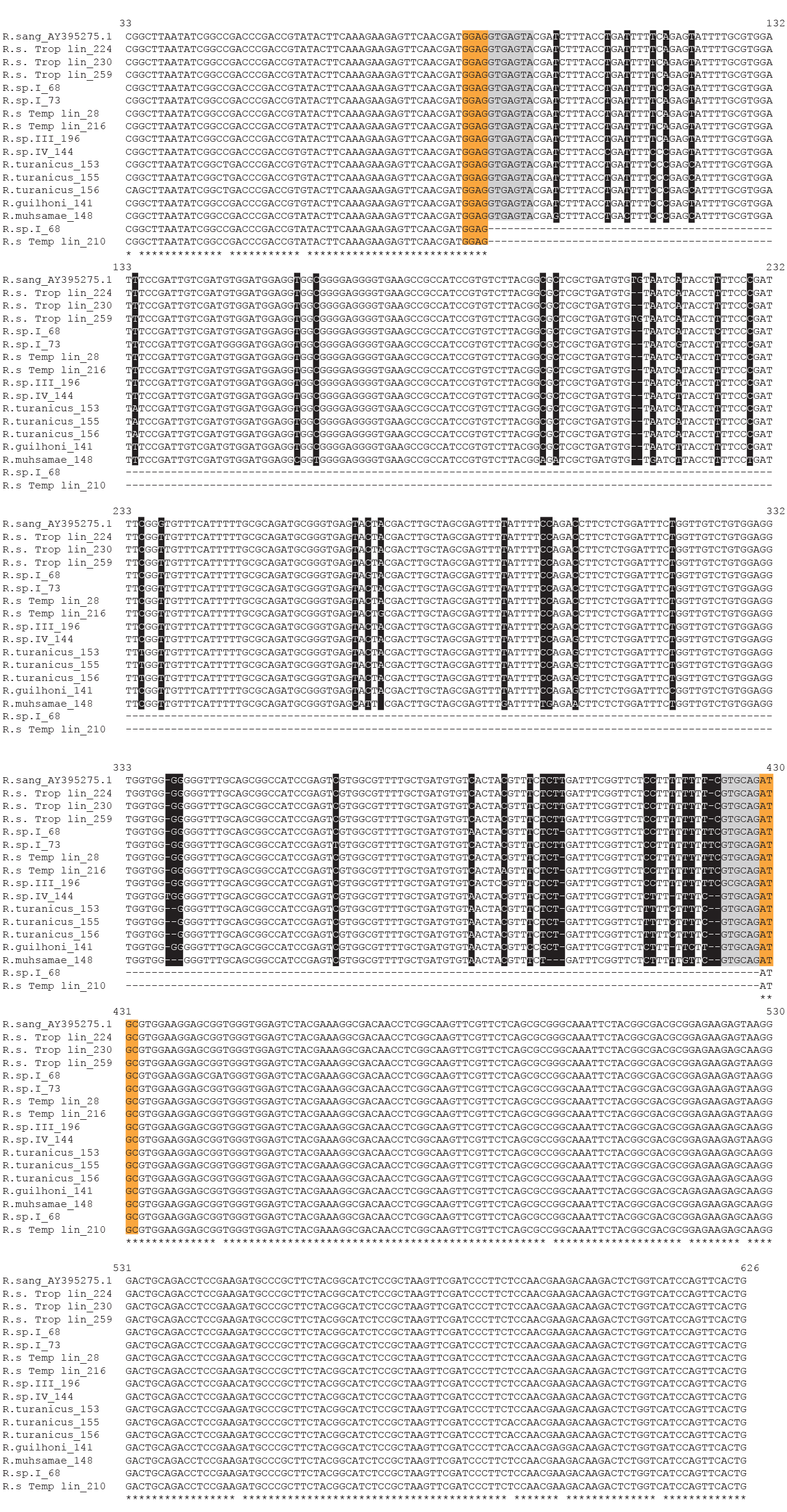

Supplement: Additional file 1: — Figure S1. Alignment of the full sequence of the crt region amplified in the Rhipicephalus spp. individuals analysed (coded as in Table 1). Nucleotides in orange boxes belong to exon regions in 5′ and 3′ splice donor regions. Nucleotides in light grey boxes belong to intron region in 5′ and 3′ splice donor regions. Nucleotides in black boxes indicate sequence identity in intron region. Asterisks show nucleotide identity in exon regions. Numbers indicate the nucleotide position of the R. sanguineus (s.l.) crt sequence AY395275 [25]. Abbreviations: R.s. Trop. lin., Rhipicephalus sanguineus (s.l.) “Tropical lineage”; R.s. Temp. lin., Rhipicephalus sanguineus (s.l.) “Temperate lineage”. (TIF 1069 kb) [file 13071_2016_1909_MOESM1_ESM.tif]
